# Clinicopathologic characteristics of thymic clear cell carcinoma: a case report with literature review

**DOI:** 10.1186/s13019-023-02150-3

**Published:** 2023-02-01

**Authors:** Zuxuan Zhao, Qingpeng Zeng, Jiangtao Li, Shan Zheng

**Affiliations:** 1 Department of Pathology, National Cancer Center/National Clinical Research Center for Cancer/Cancer Hospital, Chinese Academy of Medical Sciences and Peking Union Medical College, Beijing, 100021 China; 2 Department of Thoracic Surgery, National Cancer Center/National Clinical Research Center for Cancer/Cancer Hospital, Chinese Academy of Medical Sciences and Peking Union Medical College, Beijing, 100021 China

**Keywords:** Thymic clear cell carcinoma, Imaging, Blood test, Pathological features

## Abstract

**Background:**

Thymic clear cell carcinoma is a rare mediastinal neoplasm, with only 25 reported cases to date. We report a case of a 45-year-old man with thymic clear cell carcinoma. We think imaging and laboratory tests may be helpful for differential diagnosis.

**Case presentation:**

A 45-year-old male was admitted to a local hospital for chest distress with cardiopalmus. CT showed a mediastinal mass. Laboratory examination results were all in the normal range. Histologically, the tumor cells had a clear cytoplasm, and immunohistochemically, the tumor cells were positive for epithelial markers. We performed abdominal and pelvic CT and further examined serum levels of thyroxine, parathyroid hormone and AFP postoperatively, which were normal. The patient received postoperative radiotherapy, and CT showed left adrenal metastasis at 20 months after surgery.

**Conclusion:**

Thymic clear cell carcinoma is a rare malignant neoplasm. Adrenal metastasis can occur. Patients undergo thymectomy with chemotherapy or with radiotherapy have better outcoming. Metastasis, direct invasion of parathyroid carcinoma and other primary tumors in the mediastinum should be excluded. Immunohistochemical markers, imaging and laboratory examination can help to exclude metastasis.

## Background

Thymic clear cell carcinoma was first reported in 1983 by Wolfe et al. [1]. Here, we report a case of thymic clear cell carcinoma with adrenal metastasis. Our goals are to deepen understanding of this tumor by summarizing the clinicopathological information of this case and to propose ideas for the diagnosis and differential diagnosis of this tumor.

## Case presentation

**Fig. 1:**
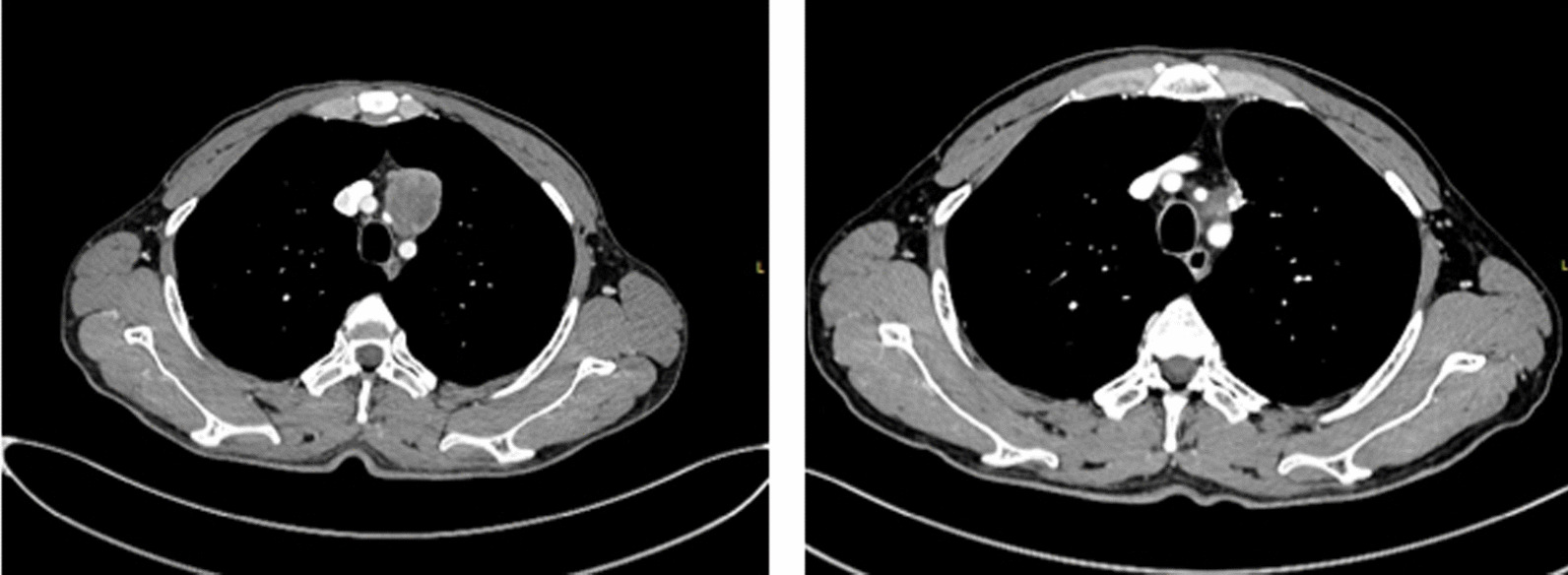
Initial enhanced CT scan of the upper mediastinum demonstrated a large anterior mediastinal mass attached to the adjacent vessels with heterogeneous enhancement (left). Post-treatment enhanced CT scan of the upper mediastinum showed residual soft tissue of the anterior mediastinum consistent with postoperative changes (right)

A 45-year-old male was admitted to a local hospital for chest distress with cardiopalmus for 1 month. Physical examination after admission revealed no abnormal findings. Initial enhanced Chest computed tomography (CT) scan of the upper mediastinum demonstrated a 4.2 * 3.4 cm large mass attached to the adjacent vessels with heterogeneous enhancement (Fig. 1). Tumor biomarkers, including CEA, CA19.9, hCG and AFP, were all within the normal range. Tracheoscopy revealed no evidence of other abnormalities. The patient underwent thoracoscopic excision of the lesion in the left superior mediastinum and part of the left lung.

**Fig. 2:**
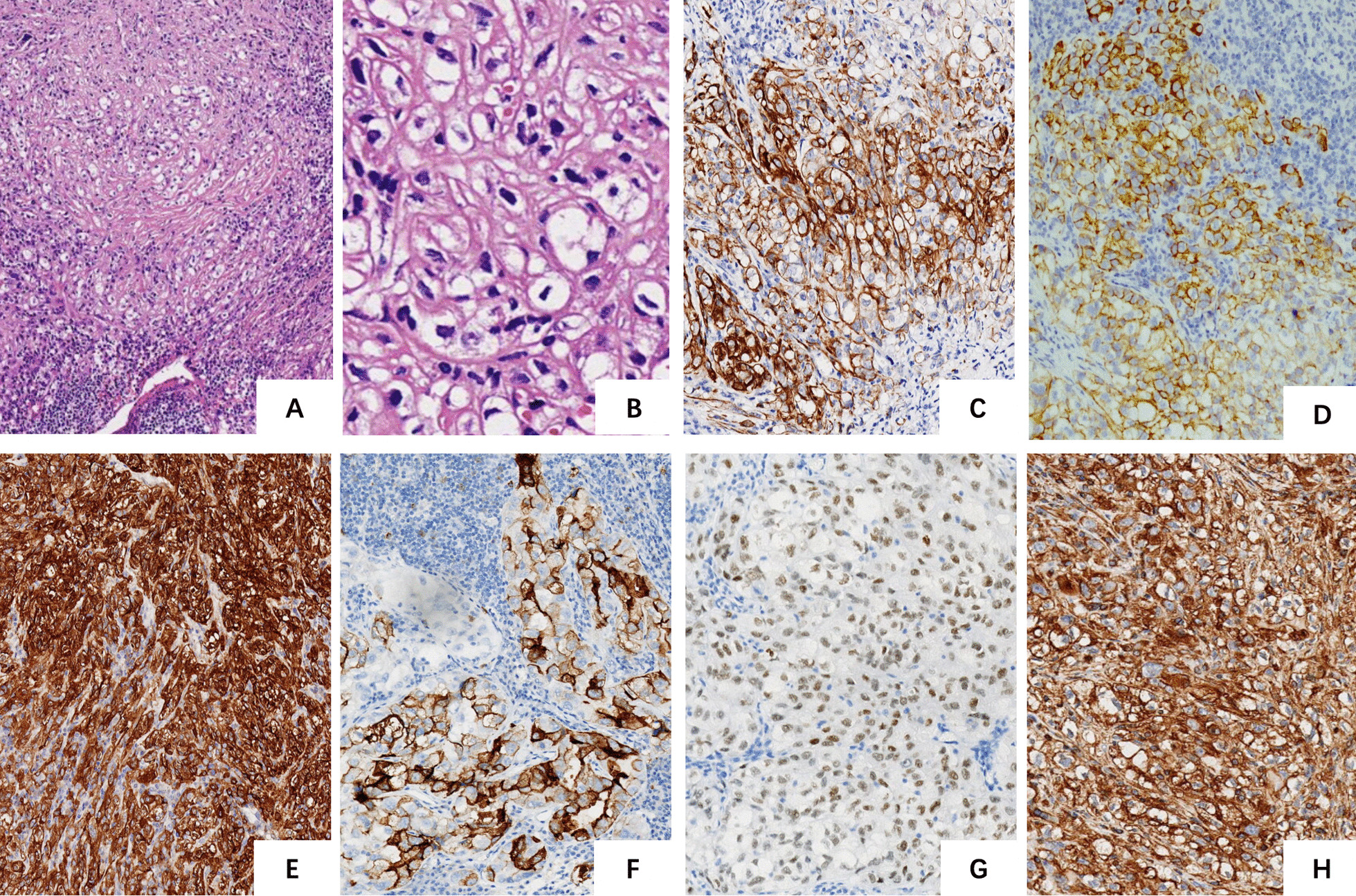
Scans of histological slides stained with hematoxylin–eosin and immunohistochemically. **a** A lobulated architecture is observed, separated by a dense fibrous stroma with lymphocyte infiltration. (4x) **b** Polygonal tumor cells with an abundant clear cytoplasm. Nuclei are round to oval with small nucleoli. (20x) **c** AE1/AE3 (10x): Note the strong diffuse positivity in the tumor cell. **d** EMA (10x): Note the positivity in the tumor cell. **e** CK18 (10x): Note the strong diffuse positivity in the tumor cell.** f** CD10 (10x): Note the partial positivity in the tumor cell. **g** PAX8 (10x): Note the strong diffuse positivity in the tumor cell. **h** Vimentin (10x): Note the strong diffuse positivity in the tumor cell

Grossly, the tumor was a firm nodular mass measuring 3.5 × 3.0 × 2.0 cm in size. The cut surface appeared grayish-white and yellow. Microscopically, the tumor showed an invasive growth pattern, which had a lobulated architecture separated by dense fibrous tissue. Hyalinized stroma can be seen in some area. Multifocal necrosis was found. The tumor cells were epithelioid and focally spindle-shaped with a predominance of clear cytoplasm; some appeared slightly eosinophilic. Immunohistochemically, the tumor cells were positive for epithelial markers (AE1/AE3, CK18, EMA) and focally expressed CD10, PAX8 and vimentin. The Ki67 index was determined to be 30% (Fig. 2). Overall, lung tumor (TTF1, NapsinA, Syno), parathyroid carcinoma (CK19, PTH), thymoma (TdT), clear cell sarcoma (HMB-45, Melanoma Pan, Melan-A), germ cell tumor (PLAP, OCT3/4, SALL4, CD30), renal tumor (RCC), salivary gland clear cell carcinoma (P63, P40) and CD117, CD5 markers were negative. Genetically, FISH results were negative for translocation of EWSR1.

We carried out further blood tests and examined his serum levels of thyroxine, parathyroid hormone and AFP postoperatively, which were normal. CT results revealed no other lesions in the kidneys. Therefore, a diagnosis of thymic clear cell carcinoma (T1bN0M0) was made according to the WHO staging system.

The patient was received regular follow-up and treated systemically with radiotherapy after surgery. CT showed no remnant in situ. However, left adrenal metastasis was occurred at 20 months after the surgery.

## Discussion and conclusions

We searched the Pubmed database and found 23 case reports thus far [1–13] (Table 1). Patients range in age from 33 to 84 years, with a median of 55 years. The male-to-female ratio is 14:9. The median survival time is 13 months (range, 4 months to 2 years).

**Table 1:** Summary of thymic clear cell carcinoma reported on Pubmed

Literature	Age/gender	Chief complaint	Immunohistochemistry and special stain	Treatment	DFS	MS
Snover et al.	42/M	Asymptomatic	PAS (+)	TT CR	108	WS
Wolfe et al.	33/M	Fever Fatigue Weight loss	PAS (+) Muci (−)	CR	0.5	Buttock
Stephens et al.	72/M	Asymptomatic	LMK (+) Muci, PAS, CEA (−)	TT CR	12*	
Kuo et al.	64/M	Dyspnea	PAS, LMK (+)	None	3	DOD
Truong et al.	69/M	Chest pain	LMK, EMA (+) Muci, Vim, PLAP (−)	TT RT	36	Lung
Hasserjian et al.	36/M	Asymptomatic	PAS, LMK, HMK, Vim (+) Muci, EMA, CEA, PLAP (−)	TT CR	24	DOD
	58/F	Asymptomatic	PAS, LMK, HMK, PLAP (+) Muci, Vim, EMA, CEA (−)	TT RT	20	DOD
	52/F	Chest pain	PAS, LMK, HMK (+) Muci, Vim, EMA, CEA, PLAP (−)	TT CR	12	Spine
	84/F	Asymptomatic	PAS, LMK, HMK (+) Muci, Vim, EMA, CEA, PLAP (−)	TT	1.5	DOD
	37/F	NA	Muci (−)	TT RT	72*	
	50/M	Chest pain	PAS (+) Muci, EMA, CEA, PLAP (−)	CR	4	DOD
	62/M	NA	PAS, LMK, HMK, EMA, CEA (+) Muci, Vim, PLAP (−)	TT RT	7*	
	36/F	Dyspnea Chest pain	PAS, LMK, HMK, EMA, PLAP (+) Muci (−)	CR	7	DOD
Okuda et al.	59/M	Dyspnea	CK, EMA (+)	CR	6*	
Nakano et al.	42/M	Asymptomatic	CK, CEA (+) Vim, PLAP (−)	TT CR	12	Brain
Lale et al.	66/F	Chest pain Dyspnea	CK7, EMA, PAS (+) CK20, Muci, P63 (−)	RT TT	NA	NA
Bertocchi et al.	36/M	Asymptomatic	CK, AE/AE3 (+) CK5/6, p63, EMA, PLAP (−)	TT CR	24*	
Dai et al.	50/F	Asymptomatic	CK, PAS, p63, CK19 (+) CK7, Vim, PLAP, Pax-8, PTH, (−)	TT CR	12*	
Porubsky et al.	66/M	NA	P40 (+) HMK, PAX8 (−)	NA	NA	NA
	52/F	NA	P40, PAX8 (+) HMK, (+)/(−)	NA	NA	NA
	64/M	NA	P40, PAX8 (+) HMK (−)	NA	NA	NA
Salgueiro et al.	61/F	Chest pain Dyspnea	AE1/AE3, CK5/6, CK7 (+)	None	2	DOD
Present case	45/M	Chest tightness	AE1/AE3, CK18, EMA, PAX8, Vimentin (+) CK19 (−)	TT RT CR	20	Adrenal gland

DFS, disease free survival; MS, metastasis site; M, male; F, female; NA, not available; TT, thymectomy; CT, chemotherapy; RT, radiotherapy; DOD, dead of disease; WS, widespread; PAS, periodic acid Schiff; LMK, low molecular weight keratin; HMK, high molecular weight keratin; EMA, epithelial membrane antigen; CK, cytokeratin; Vim, vimentin; PLAP, placental alkaline phosphatase; CEA, carcinoembryonic antigen; PAX8, paired box protein pax-8

*Disease free survival up to the last follow-up

Most chief complaints are asymptomatic (34.7%) and chest pain (26.1%) (Table 2).

**Table 2:** Descriptive statistics of the data from the case reports

Item	Mean/frequency	Percentage (%)
Cases	23	–
Age	55	–
Sex		
Male	14	60.9
Female	9	39.1
Chief complaint		
Asymptomatic	8	34.7
Dyspnea	5	21.7
Chest pain	6	26.1
Chest tightness	1	4.3
Fever	1	4.3
NA	5	21.7
Treatment		
TT + CT	7	30.4
TT + RT	6	26.1
TT	1	4.3
CT	4	17.4
None	2	8.7
NA	3	13.0
DFS		
< 12 months	6	26.1
$\geqslant$ 12 months	11	47.8
NA	6	26.1

NA, not available; TT, thymectomy; RT, radiotherapy; CT, chemotherapy; DFS, disease free survival

Clinically, the detections of mediastinal tumors mainly rely on the imaging examination, and the major method is chest CT or MRI, especially the enhanced chest CT. It can precisely show the tumor location, density, internal structure, and the relationship with the surrounding structure. MRI is easier to identify the foramen or spinal canal invasion of the tumor. Most patients underwent thymectomy with chemotherapy (30.4%) or thymectomy with radiotherapy (26.1%). According to the guideline [14], radiotherapy can be considered in patients who had capsular invasion after resection. Moreover, data improves patients underwent radiotherapy or chemotherapy after thymectomy have longer disease free survival (≥ 12 months) than patients with thymectomy alone. Different organ metastases may occur, but the adrenal metastasis in our case is firstly reported.

Microscopically, it composed of sheets, islands, and trabeculae of predominantly or exclusively of cells with optically clear cytoplasm. The nuclei are small to medium-sized with or without small nucleoli. Fibrous stroma can present, Lymphocyte infiltration is rare [15]. Hyalinizing stroma in some cases has been mentioned in 5th WHO Classification of Thoracic Tumor. It may be related to EWSR1 translocation [16]. Immunohistochemically, there is not a certain diagnostic biomarker. Tumor cells is typically positive for and PAS staining. At least one cytokeratin markers such as Low- and high- molecular-weight cytokeratin and EMA is positive. PAX8, P63 and P40 are positive in some cases [13].

For differential diagnosis, metastasis from other organs like kidney and ovary should be excluded first. Abdominal and pelvic imaging is helpful for that. Moreover, clear cell carcinoma in adjacent sites such as salivary gland and lung should also be excluded. Clear cell carcinoma of salivary gland mostly occurs in palate and base of tongue. It mostly shows distinctly hyalinized stroma and squamous differentiation. Immunohistochemically, the neoplasm always shows positivity in p63 and p40 [17]. EWSR1-ATF1 gene fusion is essential for the diagnosis [18].

After excluding distant metastasis and tumor in adjacent sites, primary mediastinal tumors characterized by clear cytoplasmic tumor cells included parathyroid adenoma/carcinoma, thymoma with clear cell components, mediastinal seminoma, among others.

The tumors mentioned above have some characteristic clinical symptoms and serological laboratory test results, which are of great help to the differential diagnosis. Parathyroid adenoma/carcinoma can have increased PTH and abnormal serum calcium and phosphorus [19]. Mediastinal seminoma can have increased β-HCG [20]. Patients with thymoma may be accompanied by systemic sclerosis [21]. Histologically, tumors mentioned above all have the features that composed of sheets, islands, and trabeculae of clear cytoplasmic tumor cells. Seminoma can have obvious nucleoli and inflammatory cell infiltration [20]. Parathyroid adenoma/carcinoma can have fine capillary network with small nucleoli [19]. Transparent components of thymoma B3 often migrate with typical thymoma regions, and bleeding and necrosis are rare [21]. There are also some characteristic immunohistochemical and molecular changes in these tumors (Table 3). Moreover, recent research shows a provisional entity in thymic carcinoma named “adamantinoma-like carcinoma”, which also has clear cell feature. However, this type of tumor is extensively desmoplastic, and has focal squamous differentiation, which may have AKT1 gene amplification [22].

**Table 3:** Differential diagnosis of primary mediastinal tumors

	Thymic clear cell carcinoma	Thymoma with clear cell component	Mediastinal seminoma	Parathyroid carcinoma with clear cell component
Median age	55	58	40	56
Clinical features	–	Accompanied with systemic sclerosis	Abnormal Β-hCG level	Abnormal PTH, serum calcium and phosphorus
Histopathology				
Common points	Islands and trabeculae of carcinoma cells with clear cytoplasm			
Different points	Sclerotic stroma often present	Transition with typical thymoma region	Large nucleoli and prominent lymphocyte infiltration	Invasive growth pattern with abundant vessels
Essential IHC results	CK (+)	TdT (T cell+)	OCT3/4, PLAP, CD117, SALL4 (+)	PTH, CK, Syn, ChrA (+)
Molecular changes	EWSR1-ATF gene infusion	–	Isochromosome 12p, 12q amplification	–

CK, cytokeratin; OCT3/4, octumer-binding transcription factor 3/4; SALL4, spalt like transcription factor 4; PLAP, placental alkaline phosphatase

EWSR1 translocation is a consistent molecular alteration in tumors with clear cell features (including clear cell carcinoma of salivary gland [18], clear cell carcinoma of lung [23], clear cell sarcoma [24]). It was firstly reported in clear cell sarcoma [24]. It was identified in cases with substantial hyalinizing stroma. As for clear cell carcinoma originating in thymus, some researchers consider that the prognoses of tumors with and without EWSR1 translocation are different. Cases with obvious hyalinized stroma with EWSR1 translocation may have better prognoses, which tend to show negativity for PAX8, CD5 and CD117. Therefore, they advocate testing for EWSR1 in cases with these histological features and adopting more conservative treatment [13].

In summary, clear cell carcinoma is a rare type of carcinoma in the mediastinum. Most patients are young or middle-aged. Imaging examination, tumor markers and hormone levels are helpful for differential diagnosis. We recommend thymectomy with chemotherapy or with radiotherapy as treatment. Histologically, tumor cells with a clear cytoplasm show an infiltrating growth pattern. Immunohistochemically, it is positive for epithelial markers. PAS staining, CK, EMA, P40, PAX8 and exclusive markers (TdT, OCT3/4, PLAP, CD117, SALL4, PTH) can be used as a diagnostic combination. EWSR1-ATF1 fusion can occur in some cases, which is related to hyalinizing stroma. Cases with EWSR1 translocation show better prognosis. Adrenal metastasis can occur, and imaging examination should focus on this site during postoperative follow-up.

## Data Availability

All data generated or analysed during this study are included in this published article.

## Abbreviations

CT: Computed tomography
AFP: Alpha-fetoprotein
CEA: Carcinoembryonic antigen
hCG: Humanchoionicgonadotophin
AE1/AE3: Pan cytokeratin
CK18: Cytokeratin 18
EMA: Epithelial membrane antigen
CD10: Cluster of differentiation 10
PAX8: Paired box protein pax-8
P63: Tumor protein P63
P40: Tumor protein P40
TTF1: Thyroid transcription factor 1
Syn: Synaptophysin
CK19: Cytokeratin 19
PTH: Parathyroid hormone
TdT: Terminal deoxynucleotidyl transferase
CD117: Cluster of differentiation 117
CD5: Cluster of differentiation 5
HMB-45: Human melanoma associated antigen 45
PLAP: Placental alkaline phosphatase
OCT3/4: Octumer-binding transcription factor 3/4
SALL4: Spalt like transcription factor 4
CD30: Cluster of differentiation 30
RCC: Renal cell carcinoma marker
FISH: Fluorescence in situ hybridization
EWSR1: Ewing sarcoma breakpoint region 1 gene
WHO: World Health Organization
